# Development of a Horseradish Peroxidase-Based Electrochemical Biosensor for Melatonin Detection

**DOI:** 10.3390/bios16090475

**Published:** 2026-08-29

**Authors:** Andra-Georgiana Trifan, Constantin Apetrei

**Affiliations:** Department of Chemistry, Physics and Environment, Faculty of Sciences and Environment, ‘Dunarea de Jos’ University of Galati, 47 Domneasca Street, 800008 Galati, Romania

**Keywords:** melatonin, biosensor, horseradish peroxidase, graphene, voltammetry

## Abstract

A horseradish peroxidase (HRP)-based electrochemical biosensor was developed for melatonin (MLT) detection using a graphene-modified screen-printed electrode (G/SPE). HRP was immobilized at different temperatures (4 °C and 20 °C) and enzyme amounts (10 and 20 µL of 5 mg/mL HRP solution) to optimize the performance of the biosensors. FTIR analysis confirmed successful enzyme immobilization, while differential pulse voltammetry (DPV) showed a clear oxidation peak of MLT at 1.1 V with enhanced current responses for HRP-modified electrodes. The optimal biosensor, prepared at 4 °C with 10 µL enzyme of 5 mg/mL HRP solution, exhibited the best analytical performance, with a linear range of 0.4–3.6 µM, and a detection limit of 1.02 µM. Kinetic studies confirmed strong enzyme-substrate affinity, while the biosensors demonstrated excellent repeatability and a good stability. The method was successfully validated on pharmaceutical products, proving to be a reliable and an accurate tool for MLT quantification in real samples.

## 1. Introduction

Melatonin (MLT) is a neurohormone synthesized primarily by the pineal gland from the essential amino acid tryptophan. Under physiological conditions, the pineal gland represents the most important source of circulating MLT in the bloodstream. MLT is produced in larger amounts during the night, and it is responsible for regulating the sleep–wake cycle [1]. In addition to its essential role in regulating circadian rhythm, MLT has several other functions in the body: antioxidant activity (MLT and its metabolites are directly involved in scavenging free radicals); a role as a modulator of lipid metabolism (particularly through the reduction in lipogenesis) and glucose homeostasis, exerting an anti-diabetic effect; the ability to reduce neoplastic spread through cytostatic and cytotoxic actions [2]; anti-amyloid activity useful in Alzheimer’s disease [3]; and beneficial activity in the treatment of Parkinson’s disease [4], brain lesions, and cerebral ischemia [5].

The detection of MLT is crucial for understanding how it inhibits cancer progression [6], how the circadian rhythm functions, and for identifying potential neuroendocrine and metabolic dysfunctions [4]. Chromatographic, spectroscopic, immunoassay, and electrochemical methods are among the many analytical techniques that have been developed for MLT detection. Each of these techniques has distinct advantages and limitations in terms of sensitivity, selectivity, analysis time, and instrumentation requirements (Table 1).

Biosensors are essential in this context, serving as valuable tools for monitoring MLT levels and for a more precise and efficient evaluation of physiological conditions. This is due to their high sensitivity, specificity, and ability to provide rapid, real-time data and point-of-care (POC) testing. Biosensors are analytical instruments that combine biological recognition elements (DNA sequences, enzymes, antibodies, etc.) with physical transducers for the specific detection of an analyte, relying on remarkable selectivity and sensitivity. Electrochemical biosensors possess a notable capacity for analyte detection due to catalytic processes occurring at the working electrode surface, which generate measurable electrochemical signals. Potentiometric, amperometric, conductometric, impedimetric, and voltammetric methods represent the primary electrochemical techniques used for the design, calibration, and optimization of electrochemical sensors and biosensors [16].

Enzymatic biosensors (e.g., horseradish peroxidase (HRP), also used in this study) have transformed many industrial applications by offering a precise, rapid, and cost-effective way to identify and measure specific analytes [17]. By utilizing the specificity of enzymes to catalyze interactions with target molecules, these devices convert biological events into quantifiable signals [18].

According to our research, no enzymatic biosensors for MLT detection have been identified in the existing literature; however, HRP-based electrochemical biosensors have been successfully reported for the detection of other compounds of interest. For example, HRP has been immobilized on different electrode platforms incorporating nanostructured materials such as graphene oxide, multi-walled carbon nanotubes, nanoporous gold, and gold nanoparticles, enabling the electrochemical detection of hydrogen peroxide, caffeic acid, dopamine, nitrite, glutathione, and sulfide using cyclic voltammetry (CV), differential pulse voltammetry (DPV), and amperometry [19,20,21,22,23,24,25,26]. HRP exhibits great versatility and can be successfully utilized to develop high-sensitivity electrochemical biosensors for analytes with diverse chemical structures, including both inorganic and organic compounds. In this context, HRP-based biosensors can be investigated regarding their potential to expand their applicability for MLT detection.

This study presents a comparative investigation of graphene-based biosensors (G/SPE) modified with HRP, monitoring the influence of the amount of enzyme deposited (10 µL and 20 µL of 5 mg/mL HRP solution) and the deposition temperature (20 °C and 4 °C). Accordingly, the following biosensors were fabricated: at room temperature with 10 µL of 5 mg/mL HRP solution (R10-HRP/G/SPE); at room temperature with 20 µL of 5 mg/mL HRP solution (R20-HRP/G/SPE); at refrigerator temperature with 10 µL of 5 mg/mL HRP solution (F10-HRP/G/SPE); and at refrigerator temperature with 20 µL of 5 mg/mL HRP solution (F20-HRP/G/SPE).

The aim of this study is the development and evaluation of an HRP-based enzymatic biosensor for MLT detection, using a rapid, sensitive, and cost-effective analytical method. The study aims to quantify MLT by leveraging the enzyme’s catalytic capacity in its reaction with the analyte, through a redox process in which electron transfer is converted into a quantifiable signal proportional to the analyte concentration.

The novelty of this study lies in the development and use of HRP-based enzymatic biosensors for the detection of MLT, a field that remains insufficiently explored. Following the literature review, no studies were identified to date on HRP-based biosensors for MLT detection, although such systems have been successfully applied for the detection of other compounds. Thus, this study proposes a new approach by extending the applicability of HRP to a new analyte, contributing to the development of alternative, efficient, and cost-effective methods for MLT determination.

## 2. Materials and Methods

### 2.1. Reagents

MLT was extracted from pharmaceutical products available on the pharmaceutical market, and the purity of the obtained compound was evaluated and confirmed using the analytical methods described in a previous article [13]. A standard 2 × 10^−3^ M MLT was prepared in 0.1 M phosphate buffer (PBS) at pH 7.0, composed of Na_2_HPO_4_ and NaH_2_PO_4_ (Sigma-Aldrich, St. Louis, MO, USA). The pH-ul was adjusted using H_3_PO_4_ or NaOH (Sigma-Aldrich, St. Louis, MO, USA) with a WTW pH meter (Xylem Analytics Germany GmbH, Weilheim, Germany) to ensure accurate measurement. Ultrapure water obtained using a Millipore Milli-Q system (KGaA, Darmstadt, Germany) with a conductivity of 18.2 MΩ·cm was used for PBS preparation. For the three-electrode electrochemical cell, a 0.1 M KCl (Sigma-Aldrich, St. Louis, MO, USA) solution was used as the salt bridge for the reference electrode, ensuring good conductivity and a stable potential. To fabricate the enzymatic biosensors, an aqueous solution of 5 mg/mL HRP (50–150 units/mg solid, Sigma-Aldrich, St. Louis, MO, USA) was prepared, and the cross-linking was achieved using glutaraldehyde 25 wt.% in H_2_O (Sigma-Aldrich, St. Louis, MO, USA). Serotonin, dopamine, uric acid, alanine, pyridoxine, ascorbic acid, and glucose purchased from Sigma-Aldrich were used for the interference study.

### 2.2. Apparatus

A Partner AS 220/C/2 analytical balance (Radwag, Radom, Poland) was used for weighing, while an Elmasonic S10H ultrasonic bath (Carl Roth GmbH, Karlsruhe, Germany) was employed for compound solubilization. The pH was adjusted using a WTW pH meter (Xylem Analytics Germany GmbH, Weilheim, Germany). The functional groups of HRP were identified via FTIR spectroscopy using a Bruker ALPHA FT-IR spectrometer (BrukerOptik GmbH, Ettlingen, Germany). Electrochemical analyses were performed in a 50 mL electrochemical cell (Princeton Applied Research, Oak Ridge, TN, USA) equipped with a three-electrode system (Ag/AgCl reference electrode, platinum counter electrode, and the working electrode-HRP/G/SPE), using a potentiostat/galvanostat (Bio-Logic Science Instruments SAS, Claix, France) and the EC-LAB EXPRESS software (Bio-Logic Science Instruments SAS, Claix, France). The graphene screen-printed electrodes were purchased from Metrohm DropSens (Metrohm DropSens S.L.U., Llanera, Spain).

### 2.3. Enzyme Immobilization and FTIR Characterization of the Biosensors

HRP immobilization on the electrodes was performed using two different volumes of HRP solution, 10 µL and 20 µL of 5 mg/mL HRP solution, to evaluate the influence of enzyme loading on the biosensor’s performance. Immobilization was achieved through cross-linking with 25% glutaraldehyde by exposing the electrodes to vapors for 60 s within a 2 cm^3^ chamber. Enzyme deposition was carried out under two temperature conditions: 20 °C and 4 °C, to investigate the effect of deposition conditions on the activity of the immobilized enzyme.

This comparative approach allows for the assessment of the impact of both enzyme quantity and immobilization conditions on the electrochemical performance of the biosensor, with testing conducted via cyclic voltammetry for MLT detection. Thus, the objective is to identify the most efficient conditions for developing a sensitive and stable biosensor capable of detecting MLT in biological samples. The biosensors were characterized by FTIR spectroscopy to confirm the presence of the immobilized enzyme on their surface.

### 2.4. Electrochemical Characterization of the Biosensor and Enzymatic Kinetics Analysis

By using the standard addition method, different volumes of MLT standard solution were added to the electrochemical cell into a solution consisting of 50 mL PBS (0.1 M, pH 7.0) and 1 mL 3% H_2_O_2,_ resulting in MLT concentrations ranging from 0.4 µM to 413 µM. Differential pulse voltammetry (DPV) was employed as the analytical method. The voltammograms corresponding to each MLT concentration were recorded, and the obtained anodic and cathodic current values were used to construct the calibration curves. The limits of detection (LOD) (1) and quantification (LOQ) (2) [12] were calculated based on the standard deviation of the current measured in the blank sample and the slope of the calibration curve.

LOD = 3 × σ/slope(1)

LOQ = 10 × σ/slope(2)

where:

- LOD = limit of detection;
- LOQ = limits of quantification;
- σ = standard deviation.

The kinetic analysis of the enzymatic biosensor was performed to determine the kinetic parameters specific to the interaction between the enzyme and the substrate under controlled electrochemical conditions. Enzymatic kinetics were evaluated by recording the anodic current at different MLT concentrations, and the kinetic parameters, maximum current (I_max_) and apparent Michaelis–Menten constant ($K_{m}^{app}$) were calculated based on the Michaelis–Menten model using the response current values as a function of substrate concentration [27]. The apparent Michaelis–Menten constant ($K_{m}^{app}$) represents the substrate concentration at which the enzymatic reaction rate reaches half of its maximum value and is also one of the most important kinetic parameters. Data analysis was carried out using the Lineweaver–Burk equation (3), adapted for biosensors [28].

(3) $$\frac{1}{I}=\frac{1}{I_{max}}+\frac{K_{m}^{app}}{I_{max}}\times \frac{1}{\left[S\right]}$$

where:

- I = measured current, A;
- [S] = substrate concentration, M;
- $K_{m}^{app}$ = apparent Michaelis–Menten constant, M;
- I_max_ = maximum current, A.

## 3. Results and Discussion

### 3.1. FTIR Characterization

**Figure 1:**
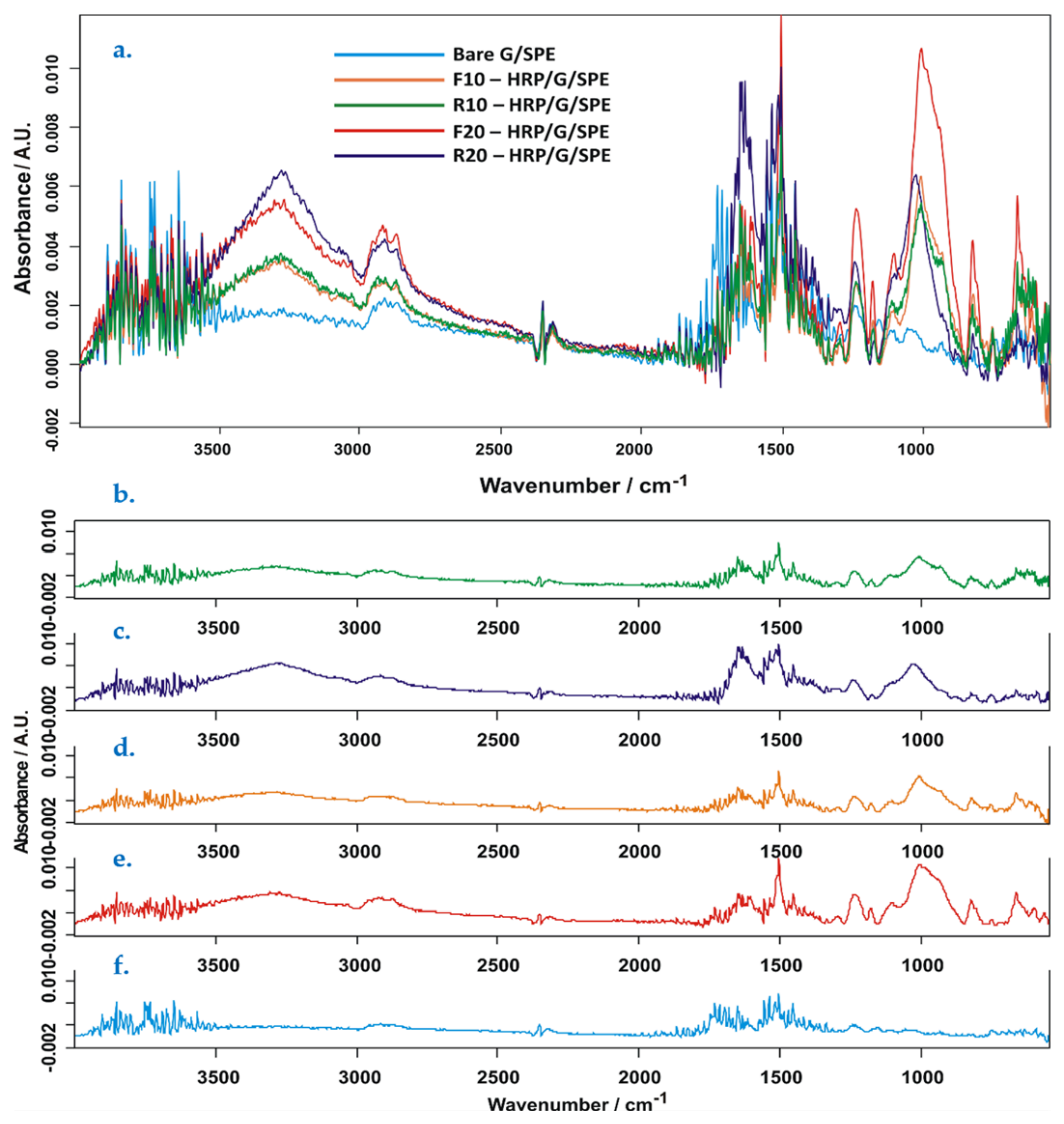
FTIR spectra of the G/SPE sensor and the R10-HRP/G/SPE, R20-HRP/G/SPE, F10-HRP/G/SPE, and F20-HRP/G/SPE biosensors: (**a**) overlaid; (**b**) R10-HRP/G/SPE; (**c**) R20-HRP/G/SPE; (**d**) F10-HRP/G/SPE; (**e**) F20-HRP/G/SPE; (**f**) bare G/SPE.

To monitor the HRP immobilization process, the FTIR spectra of the biosensors (Figure 1) were analyzed. The broad band observed in the approximate region of 3200–3400 cm^−1^ is attributed to the O-H and N-H stretching vibrations, characteristic of hydroxyl and amino groups within the amino acid structure and peptide bonds. In the 2850–2950 cm^−1^ region, bands corresponding to C-H stretching vibrations of aliphatic groups (-CH_2_ and -CH_3_) present in the amino acid side chains can be observed [29]. An intense peak around 1650 cm^−1^ is characteristic of the amide I band, associated with the C=O stretching vibration of the peptide bond, indicating the protein structure of HRP. Additionally, the band around 1540 cm^−1^ corresponds to the amide II band, resulting from the combination of N-H bending and C-N stretching vibrations [30].

In the 900–1300 cm^−1^ range, bands associated with C-N and C-O vibrations appear, originating from the amino acid structure and possible carbohydrate components of the glycoprotein [31]. The higher intensity of the peaks observed for the R20-HRP/G/SPE and F20-HRP/G/SPE biosensors is attributed to the larger amount of enzyme deposited. Therefore, the FTIR spectra confirm the presence of functional groups characteristic of the HRP structure.

The efficient immobilization of the enzyme on the electrode surface is supported by the FTIR results, which confirm the presence of functional groups specific to HRP, as well as by the subsequent reproducible and sensitive electrochemical response of the biosensors. The correlation between these results indicates the formation of an active and stable enzymatic layer deposited on the graphene electrode surface.

### 3.2. The Influence of Immobilization Temperature and the Amount of Deposited Enzyme

**Figure 2:**
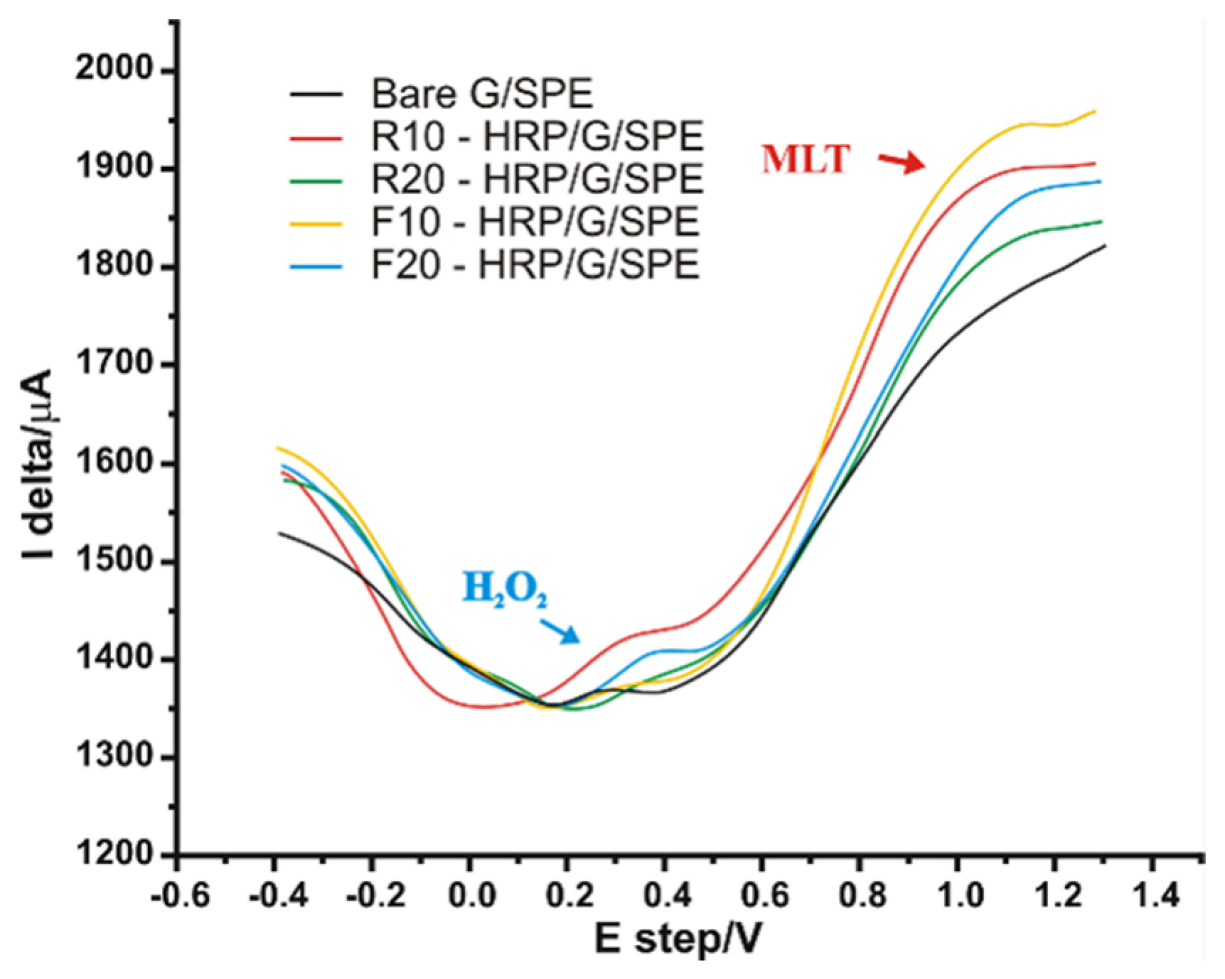
Overlaid DPV voltammograms of the modified HRP/G/SPE biosensors and the G/SPE sensor, immersed in a 2 × 10^−3^ M MLT stock solution.

Figure 2 shows the DPV voltammograms of the modified HRP/G/SPE biosensors immersed in the MLT stock solution, compared to the unmodified G/SPE sensor. A well-defined anodic peak is observed at a potential of 1.1 V, associated with the oxidation of MLT. In the case of the biosensors, the peak intensity is higher than that of the unmodified sensor, confirming that the presence of HRP leads to a significant increase in current due to the catalytic process and the facilitation of electron transfer between the analyte and the electrode [32]. The F10-HRP/G/SPE biosensor exhibits the highest peak intensity (1946.28 µA), while the F20-HRP/G/SPE biosensor shows the lowest peak intensity (1827.32 µA) among all biosensors. The peak observed at 0.3 V is attributed to the oxidation of H_2_ O_2_ present in the solution [33].

**Figure 3:**
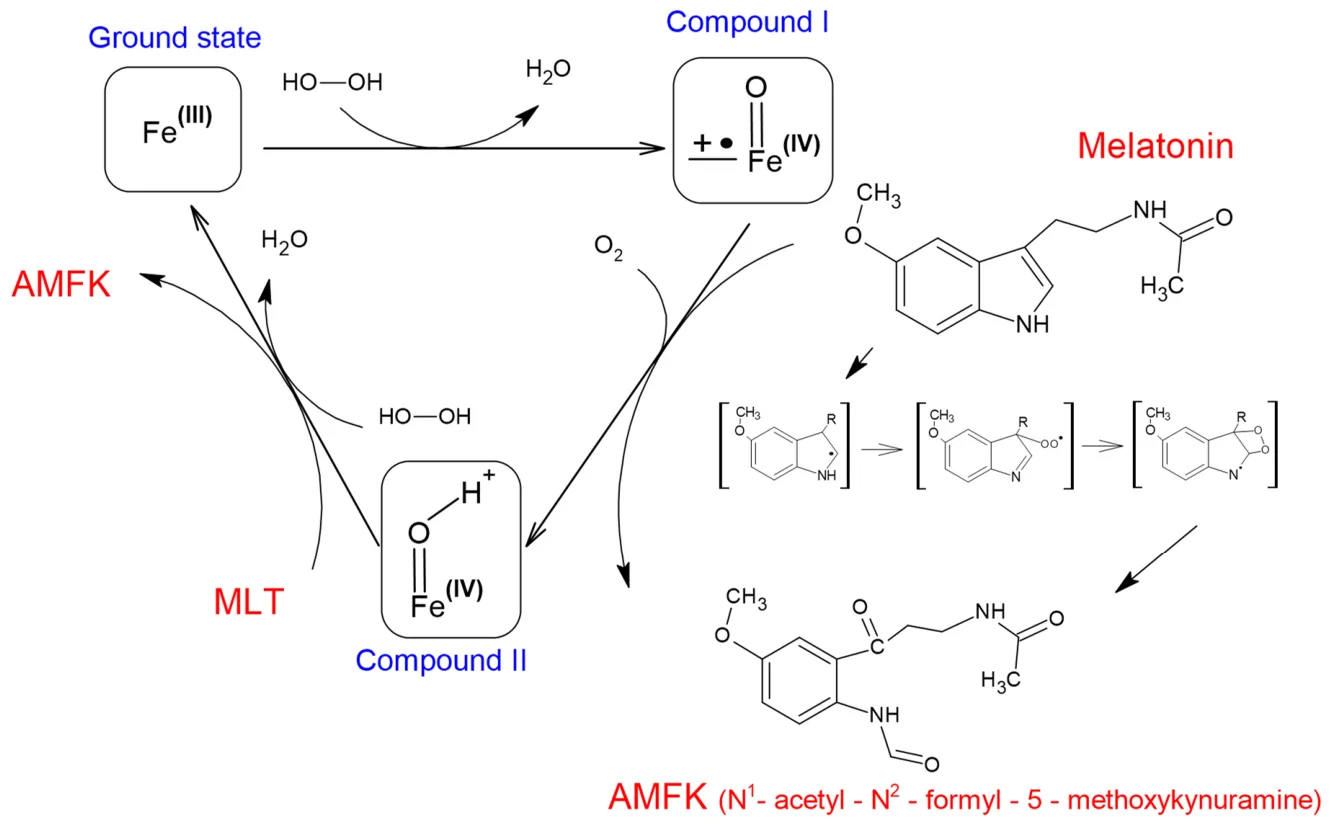
Possible oxidation reactions of MLT in the presence of HRP as a biocatalyst.

The role of HRP immobilized on the sensitive layer of the biosensors in the voltammograms could be explained as follows. HRP is an enzyme from the oxidoreductase class, containing a heme group in its active center and uses hydrogen peroxide as an electron acceptor [34]. HRP has a mechanism of action involving redox intermediates, which has been studied through several methods [35,36,37,38]. In this process, the enzyme can accept electrons provided by MLT, which acts as an antioxidant agent [38,39]. This mechanism can serve as the basis for MLT detection using biosensors that incorporate HRP as the recognition element (Figure 3).

The oxidation of indolic compounds by HRP generally involves the native enzyme-Compound I-Compound II catalytic cycle. The interaction between H_2_O_2_ and the heme group of native HRP leads to the reduction of H_2_O_2_ to H_2_O and the formation of Compound I, a ferryl π-cation radical complex (Fe(IV)=O) containing an oxygen atom generated from the cleavage of hydrogen peroxide [28]. Compound I exhibits strong oxidative capability and can oxidize MLT through a single-electron transfer mechanism [40]. In this step, MLT is oxidized into its main oxidation product, N^1^-acetyl-N^2^-formyl-5-methoxykynuramine (AMFK), with the additional consumption of atmospheric oxygen [38,41]. Compound I is thereby reduced to Compound II, which also contains a ferryl group. Although Compound II is generally less reactive, it is also able to catalyze MLT oxidation in the presence of H_2_O_2._ At the end of the reaction, the active site returns to its native form, completing the HRP biocatalytic cycle [34].

**Figure 4:**
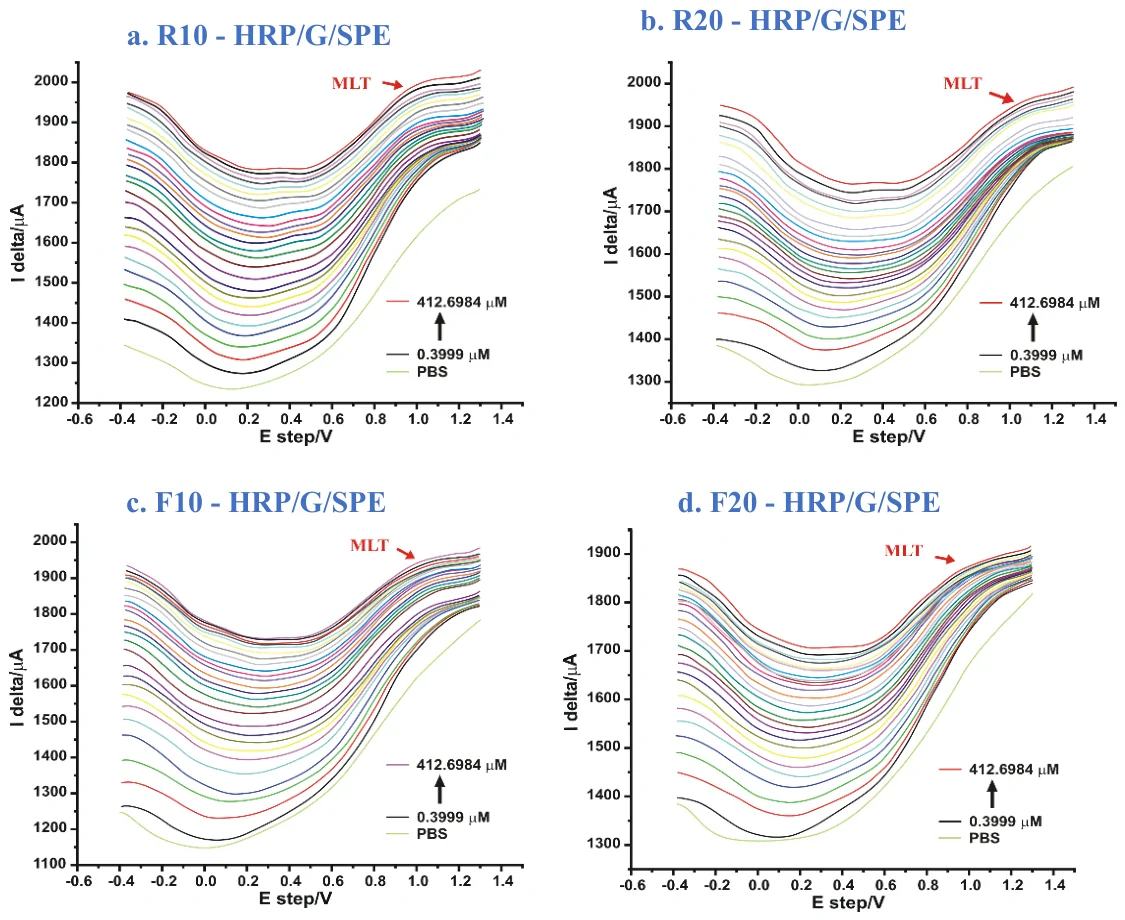
Overlaid voltammograms of the (**a**) R10-HRP/G/SPE, (**b**) R20-HRP/G/SPE, (**c**) F10-HRP/G/SPE, and (**d**) F20-HRP/G/SPE biosensors immersed in MLT solutions of different concentrations.

**Figure 5:**
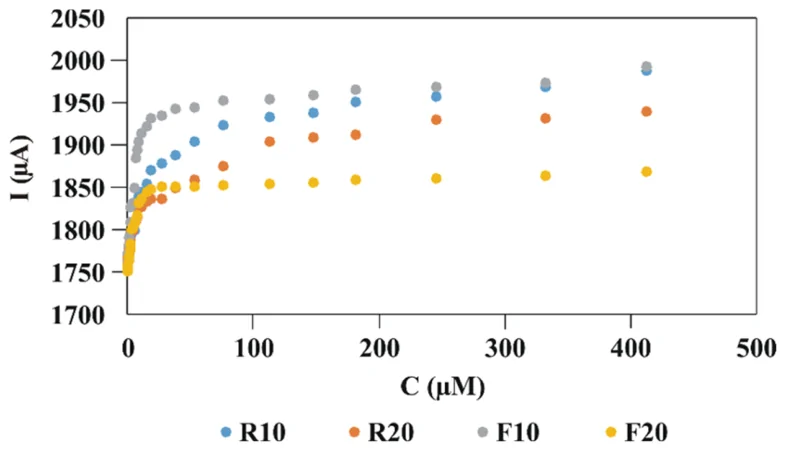
Dependence between current and MLT concentration of the R10-HRP/G/SPE, R20-HRP/G/SPE, F10-HRP/G/SPE, and F20-HRP/G/SPE biosensors.

**Figure 6:**
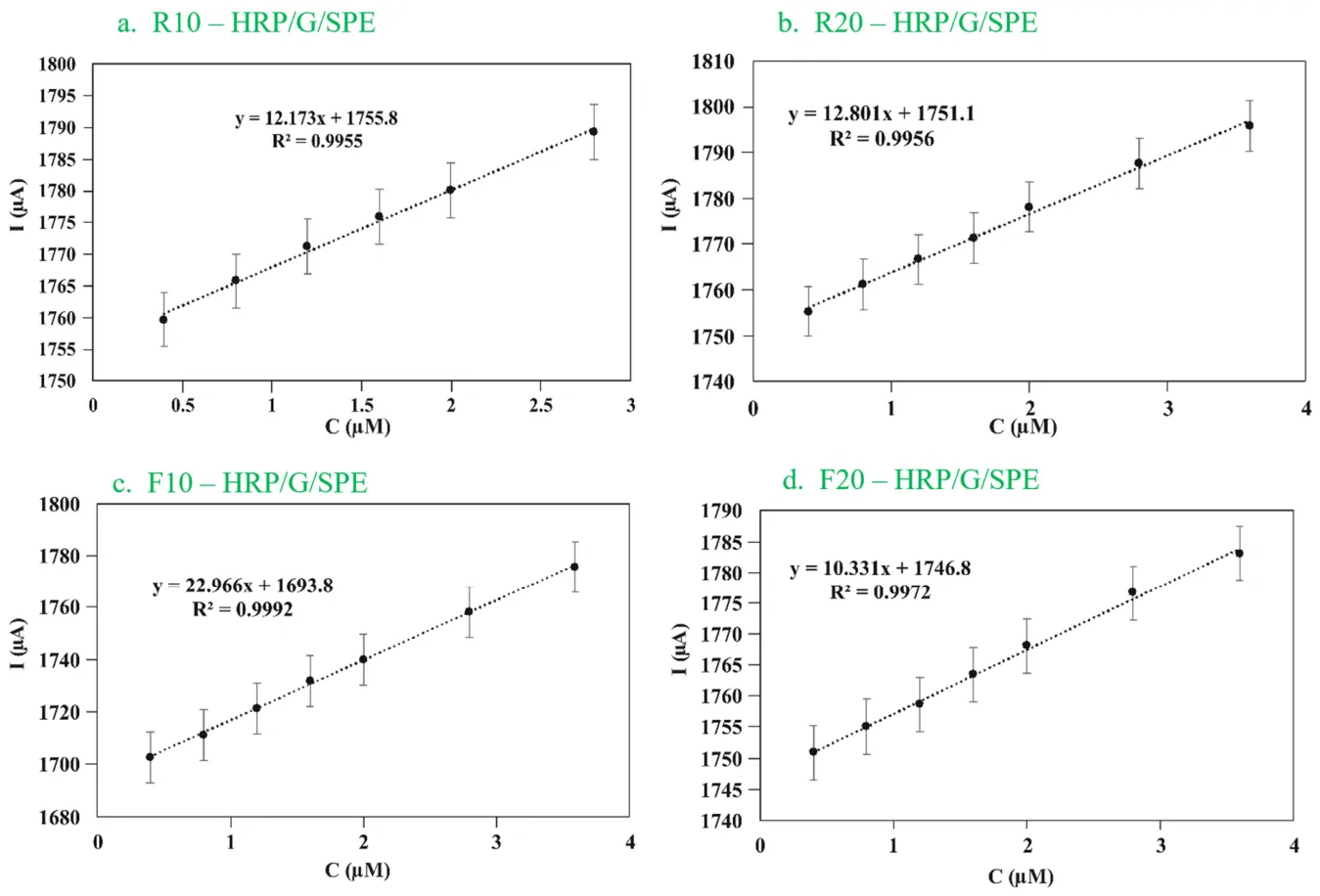
Calibration curves of the (**a**) R10-HRP/G/SPE, (**b**) R20-HRP/G/SPE, (**c**) F10-HRP/G/SPE, and (**d**) F20-HRP/G/SPE biosensors detecting MLT.

For the development of calibration curves of all biosensors developed in this study the responses of biosensors were registered with DPV in MLT of different concentrations. The current intensity increases proportionally with MLT, concentration, indicating a process controlled by electron transfer associated with the redox reaction catalyzed by HRP (Figure 4, Figure 5 and Figure 6). However, at higher concentration levels of MLT the responses of biosensors slowly increase indicating the achievement of steady state current. The concentration ranges where the current linearly increases with the concentration were used for the development of linear models. The linear dependence observed between the electrochemical signal and the analyte concentration demonstrates the viability of the biosensor for MLT quantification.

The analytical performance of the developed biosensors was assessed through the determination of the limits of detection (LOD) and quantification (LOQ). The R10-HRP/G/SPE biosensor exhibited an LOD of 1.77 μM and an LOQ of 5.90 μM (linear range 0.4–2.8 μM), while the R20-HRP/G/SPE biosensor showed an LOD of 1.76 μM and an LOQ of 5.86 μM (linear range 0.4–3.6 μM). The F10-HRP/G/SPE biosensor achieved the lowest detection limit, with an LOD of 1.02 μM and an LOQ of 3.40 μM (linear range 0.4–3.6 μM). For the F20-HRP/G/SPE biosensor, the LOD and LOQ were 1.86 μM and 6.19 μM, respectively (linear range 0.4–3.6 μM).

For the HRP/G/SPE biosensor, immobilization at 4 °C led to optimal performance with 10 µL of 5 mg/mL HRP solution, confirming that low temperatures can better preserve the enzyme’s structure and activity. However, at higher amounts (20 µL of 5 mg/mL HRP solution), the effect was reversed and sensitivity decreased. The deposition of thicker enzymatic layers can hinder electron transfer and substrate diffusion toward the active site.

Temperature conditions play a vital role in the stability and activity of HRP. Low temperatures reduce the risk of enzyme denaturation during the immobilization process and preserve the structural integrity of HRP, thereby ensuring higher post-immobilization enzymatic activity [27].

The HRP/G/SPE biosensor demonstrates high sensitivity when immobilization is performed at low temperatures and with an optimal enzyme volume. This performance is attributed to the large active surface area and high conductivity of graphene [42], which facilitate rapid electron transfer and maintain the catalytic activity of the enzyme.

The developed biosensor offers several advantages compared with previously reported MLT sensors [12,13,14,15], including a simple design, and an HRP-based enzymatic biorecognition mechanism. The proposed biosensor combines a competitive LOD (1.02 μM) with a straightforward fabrication and measurement procedure, without requiring sophisticated instrumentation or complex electrode modifications. These features make the developed biosensor promising for the future development of portable, disposable, and cost-effective MLT sensing devices.

### 3.3. Analysis of Enzymatic Kinetic Parameters

**Figure 7:**
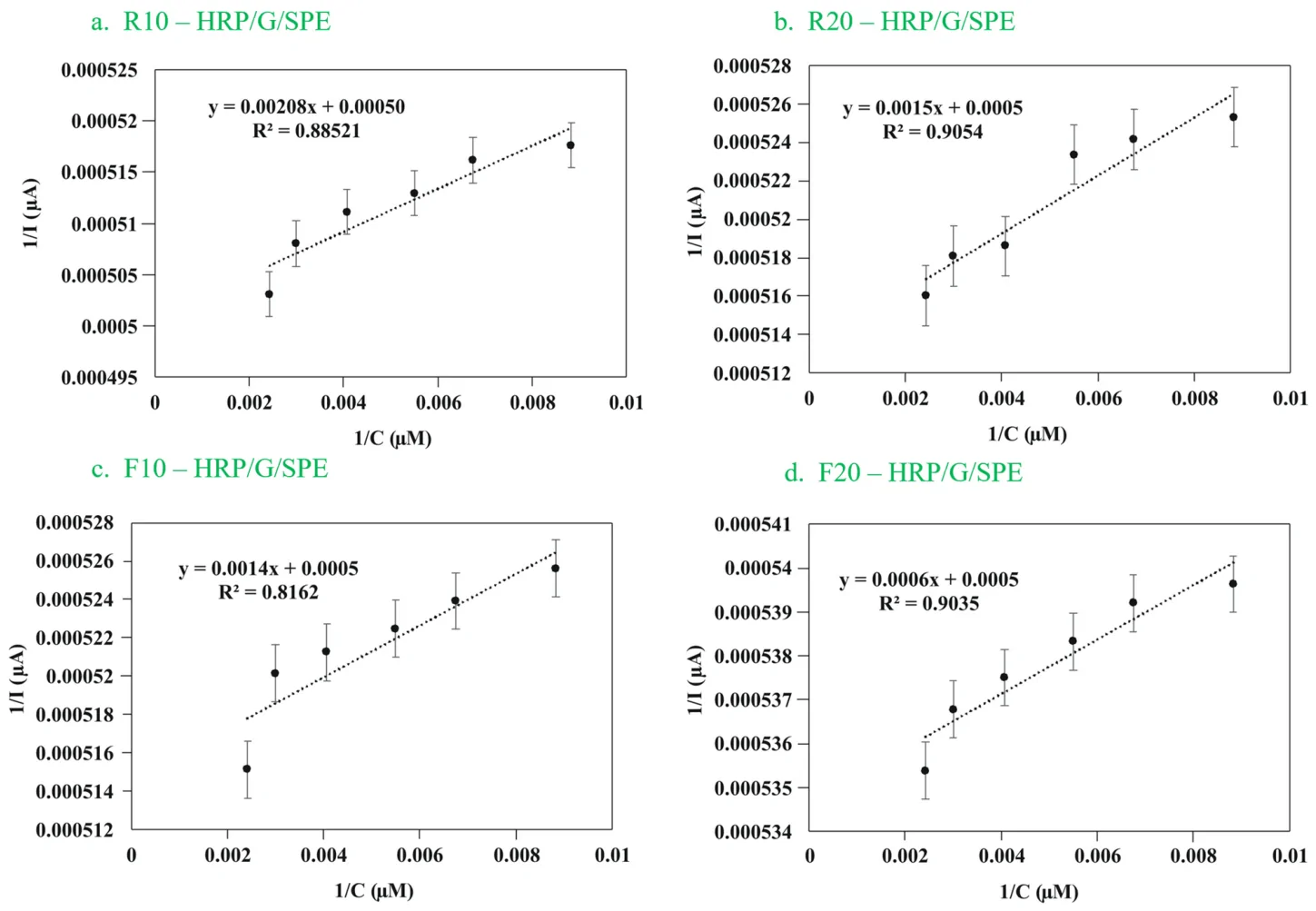
Michaelis–Menten kinetic plots of the R10-HRP/G/SPE (**a**), R20-HRP/G/SPE (**b**), F10-HRP/G/SPE (**c**), and F20-HRP/G/SPE (**d**) biosensors towards MLT solutions of different concentrations.

The enzymatic kinetic parameters obtained for the HRP/G/SPE biosensors indicate similar maximum current values (with the highest I_max_ of 2000 μA for F10-HRP/G/SPE and the lowest of 1870.55 μA for F20-HRP/G/SPE), suggesting comparable catalytic activity for all analyzed configurations and that the electrochemical response is mainly limited by electron transfer processes. Michaelis–Menten plots are provided in Figure 7. In enzymatic biosensors, electron transfer is often constrained because the redox enzyme’s active site is located within the protein structure [43].

In contrast, the values of the apparent Michaelis–Menten constant ($K_{m}^{app}$) vary depending on the amount of immobilized enzyme (Table 2). Increasing the volume of the HRP solution from 10 to 20 µL (and consequently the amount of enzyme) leads to a significant decrease in $K_{m}^{app}$ (from 4.15 µM and 2.63 µM to 2.91 µM and 1.16 µM, respectively), indicating a higher enzyme affinity for the substrate and improved catalytic efficiency [44], due to the larger amount of enzyme. Immobilization temperature (20 °C vs. 4 °C) also influences the kinetic parameters, with the best value being obtained for the biosensor prepared at 4 °C with 20 µL HRP.

The lower values obtained for the apparent Michaelis–Menten constant, compared with those reported in the literature (Table 3), indicate an increased affinity between the enzyme and the substrate, suggesting efficient immobilization and a favorable orientation of the enzyme active sites on the electrode surface [45].

### 3.4. Repeatability and Stability

**Figure 8:**
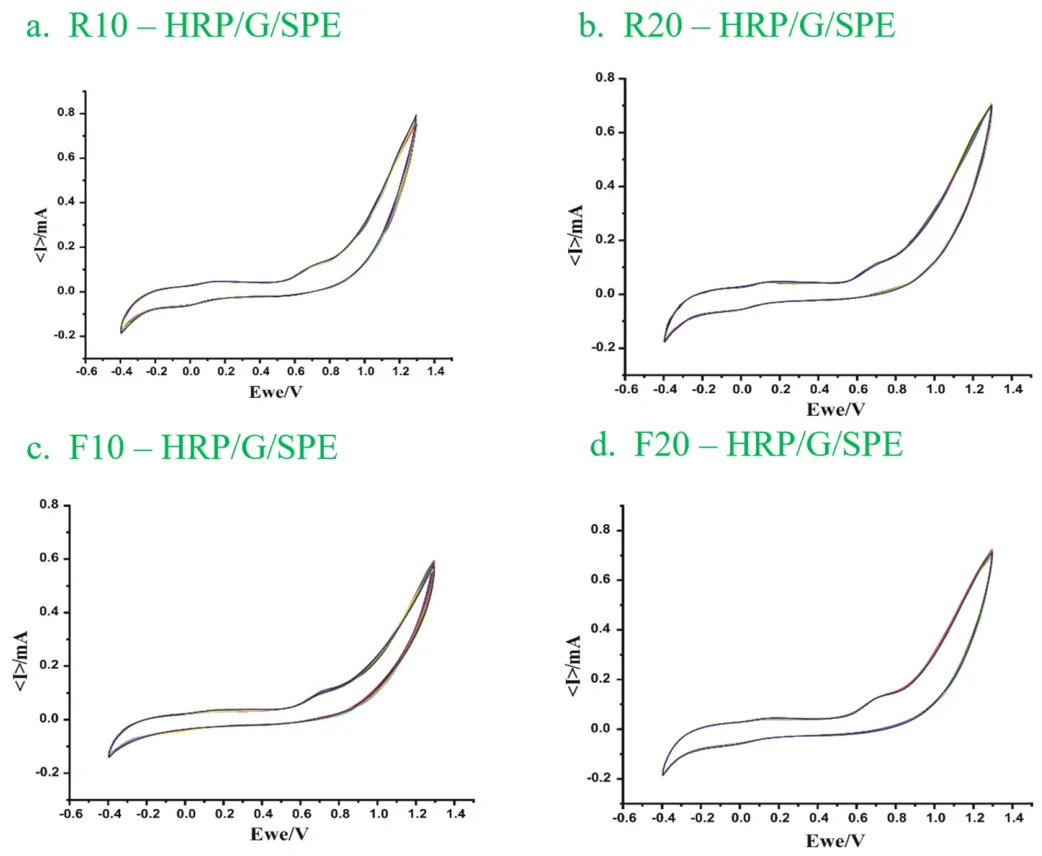
Repeatability of the biosensors evaluated by cyclic voltammetry (**a**) R10-HRP/G/SPE, (**b**) R20-HRP/G/SPE, (**c**) F10-HRP/G/SPE, (**d**) F20-HRP/G/SPE in the MLT stock solution.

The repeatability of the biosensors was evaluated by recording 10 con-secutive cyclic voltammograms in the MLT stock solution (Figure 8). The resulting coefficient of variation was 2% for all analyzed biosensors. This low variation indicates excellent reproducibility of the electrochemical response and high stability of the enzymatic layer immobilized on the electrode surface.

**Figure 9:**
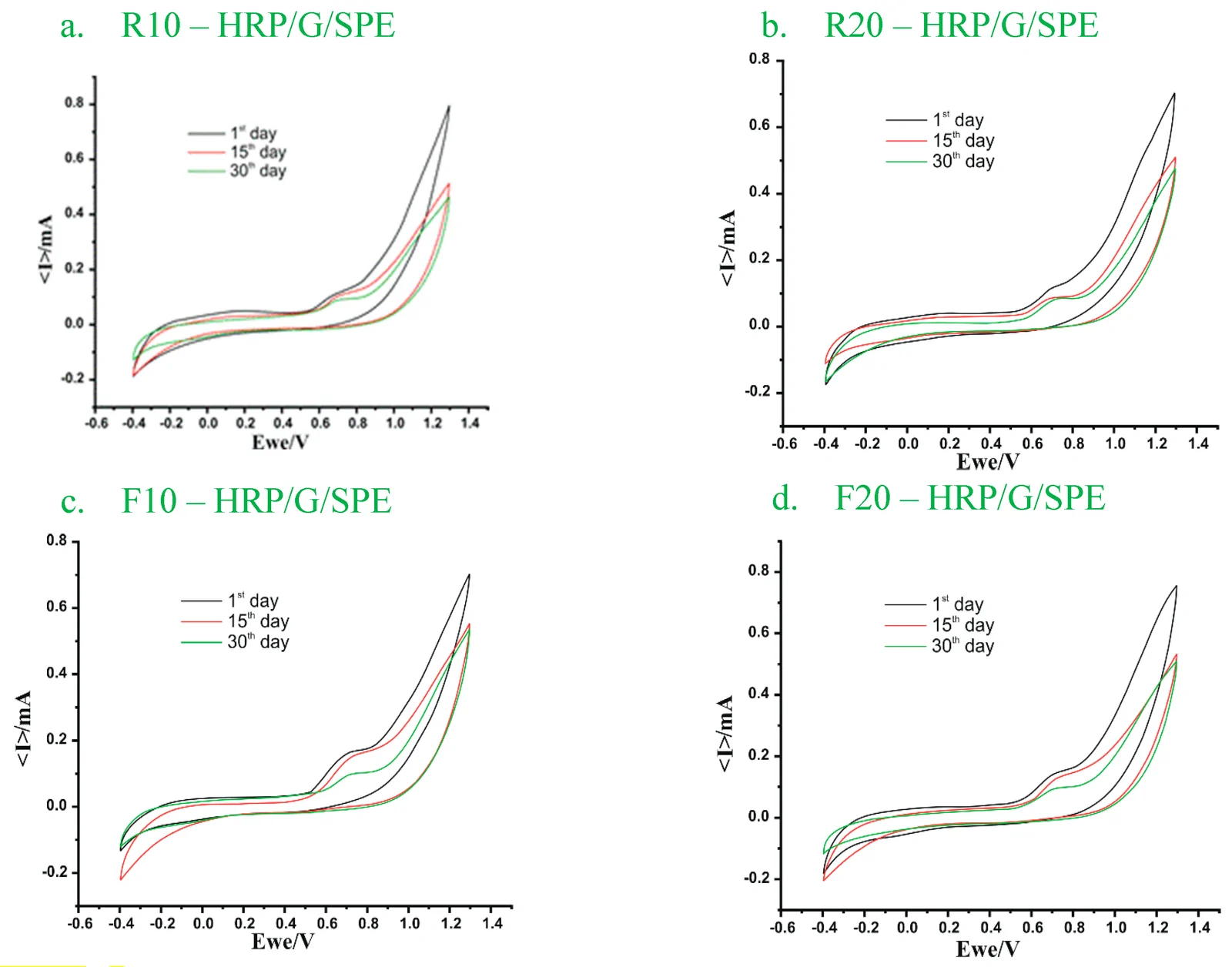
Long-term stability of the biosensors evaluated by cyclic voltammetry in MLT stock solution on the 1st, 15th and 30th day of storage (**a**) R10-HRP/G/SPE, (**b**) R20-HRP/G/SPE, (**c**) F10-HRP/G/SPE, (**d**) F20-HRP/G/SPE in the MLT stock solution.

Long-term stability was evaluated over a period of 30 days (day 1, day 15, and day 30), with the biosensors stored at 4 °C under dark conditions (Figure 9). The biosensors exhibited good structural stability throughout this period; however, a progressive decrease in anodic current intensity was observed, correlated with the decline in the activity of the immobilized enzyme. Biosensors prepared at low temperatures showed slightly superior stability (the coefficient of variation for F10-HRP/G/SPE and F20-HRP/G/SPE was 25.9% and 26.33%, respectively) compared with those prepared at 20 °C (the coefficient of variation for R10-HRP/G/SPE and R20-HRP/G/SPE was 31.23% and 39.81%, respectively), suggesting that immobilization and storage at 4 °C contribute to the formation of a more stable enzymatic layer and to the preservation of the enzyme’s native structure.

Refrigeration conditions slowed down the enzyme degradation processes, allowing the biosensors to function over the long term; however, enzymatic activity decreased progressively. To ensure measurement accuracy, it is necessary to either recalibrate the biosensors before use or utilize them within a shorter time interval.

### 3.5. Study of Interferences

The selectivity of the biosensor (F10-HRP/G/SPE) was evaluated in the presence of potential interfering compounds such as serotonin, dopamine, uric acid, alanine, pyridoxine, ascorbic acid, and glucose. The study was performed using a 2 × 10^−6^ M MLT solution, while the concentration of the interfering compounds increasing from 2 × 10^−5^ M to 2 × 10^−3^ M. Cyclic voltammograms were recorded in pure MLT solution and in MLT solution with increasing interferent concentrations. MLT recovery values were calculated when the ratio MLT: interferent was 1:1. Another calculated parameter was tolerance limit concentration, which represents the concentration of interferent that produce a 5% change in the biosensor response. The results are comprising in Table 4.

As shown in Table 4, the presence of the interfering compounds at the same level of concentration produced only minor changes in the analytical response of MLT, the recovery values being close to 100%.

The most pronounced influence was observed for serotonin, with a recovery of 95.8% (RSD of 3.1%) and tolerance limit of 3.2 × 10^−5^ M. This can be attributed to the similar electrochemical characteristics of the two molecules and to the presence of common structural features, which can facilitate competitive interactions at the electrode surface. The smallest influence was observed for uric acid, recovery of 99.8% (RSD of 2.2%) and tolerance limit of 2.0 × 10^−3^ M, a compound with different structural features and properties, less able to interact with HRP from the biosensor active surface.

The results of the interference study indicate good selectivity of the HRP biosensor for MLT, even in the presence of interfering compounds at concentrations several times higher than MLT.

### 3.6. Validation on Pharmaceutical Products

**Figure 10:**
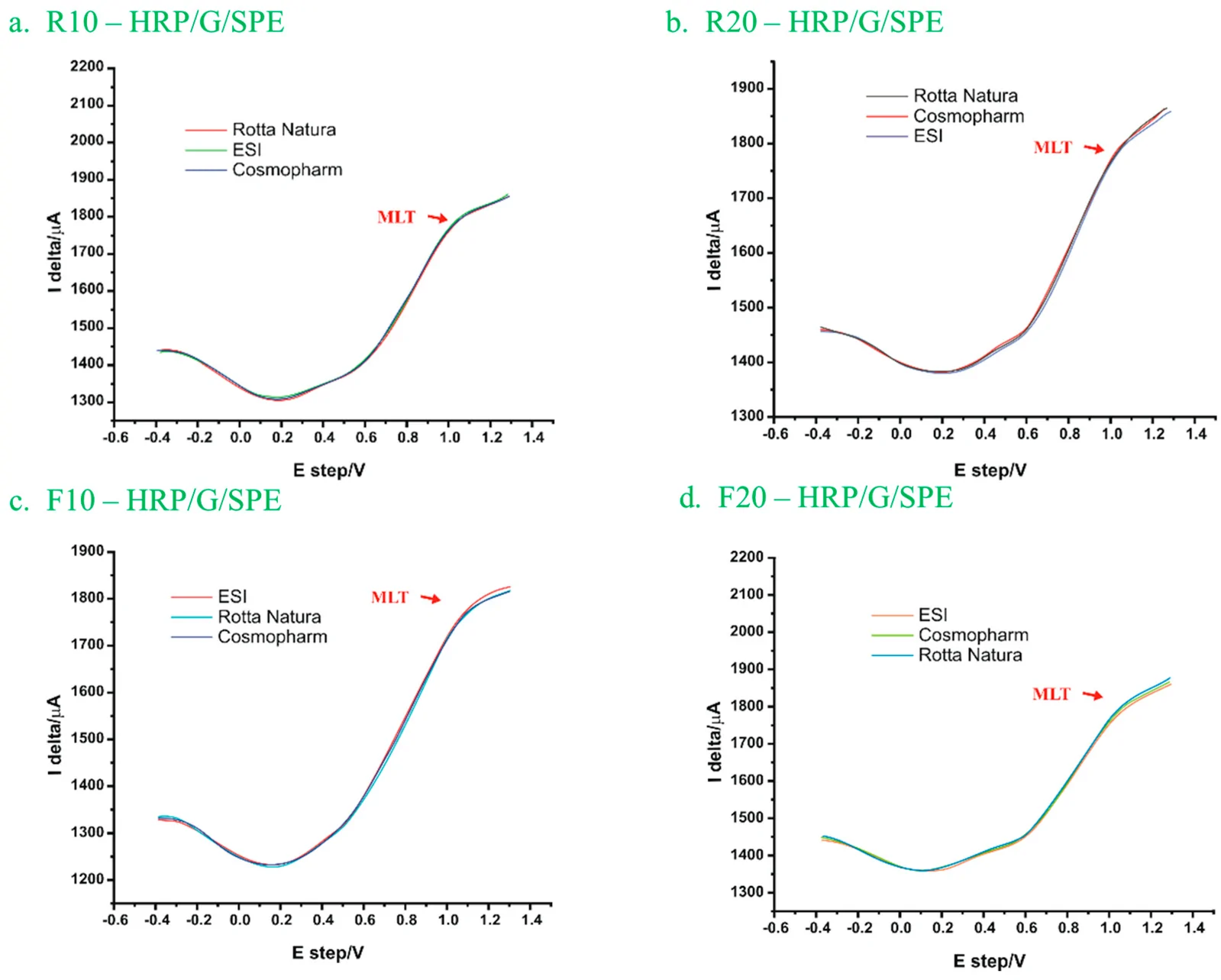
DPV voltammograms of the pharmaceutical products for each biosensor (**a**) R10-HRP/G/SPE, (**b**) R20-HRP/G/SPE, (**c**) F10-HRP/G/SPE, (**d**) F20-HRP/G/SPE.

The developed biosensors were subsequently used to quantify MLT in three pharmaceutical products (Melatonină Retard 5 mg from Rotta Natura, Melatonin Pura 5 mg from ESI, and Melatonină 5 mg from Cosmopharm). Quantification was performed using the DPV method, resulting in characteristic peaks corresponding to the presence of MLT. Representative DPV voltammograms recorded for pharmaceutical samples are shown in Figure 10. The MLT concentration in these products was calculated using the calibration curve of each biosensor.

Table 5 presents a comparison between the theoretical and experimental values of signal intensities and concentrations for the Rotta Natura, ESI, and Cosmopharm products, obtained using the four biosensors. The experimental intensity values are close to the theoretical ones, indicating a strong correlation between the calculated and experimental results. This correspondence is also reflected in the analytical recovery values, which are approximately 100%, indicating the high accuracy of the method employed.

## 4. Conclusions

Low temperature (4 °C) during immobilization better preserved the structural integrity and enzymatic activity. The optimal biosensor was identified as the one prepared at 4 °C with an optimal enzyme volume of 10 µL (F10-HRP/G/SPE), which exhibited the lowest detection limit. The biosensors showed high sensitivity and a linear dependence between the electrical signal and melatonin concentration. A very high affinity between the enzyme and the substrate was observed, as demonstrated by the low Michaelis–Menten constant values. The method was successfully validated using real pharmaceutical products, achieving an analytical recovery of approximately 100%, which confirms the high accuracy of the biosensors for melatonin monitoring. Although refrigeration conditions extend the usable lifetime of the biosensors, their enzymatic activity gradually decreases over time, with a coefficient of variation of 25.9% after 30 days, necessitating their use within a relatively short period.

## Figures and Tables

**Table 1:** Comparison of some analytical methods reported for MLT detection.

Detection Technique	Sample	Linear Range/LOD	Main Advantages	Limitations	Reference
LC-MS/MS	Human urine	7.5–500 pg/mL	High selectivity and sensitivity; simultaneous determination of melatonin and 6-hydroxymelatonin; suitable for biological samples	Sophisticated and expensive instrumentation	[7]
LC-MS/MS	Human saliva	2.15–430 pmol/L/0.43 pmol/L	High sensitivity and specificity; simultaneous determination of melatonin and cortisol	Sophisticated instrumentation and sample extraction	[8]
Colorimetry	Human saliva	2.5–38 µM/ 1.3 µM	Simple, rapid and visually detectable; good recovery	Chemical derivatization and optimized reaction conditions; relatively poor sensitivity for low MLT concentrations	[9]
Fluorometry	Human saliva	0.01–0.1 µM/ 0.004 µM	Higher sensitivity than colorimetry and good recovery	Fluorescence instrumentation and chemical derivatization, limiting simplicity and POC applicability	[9]
Indirect competitive ELISA	Health products	0.05–1.3 ng/mL/ 0.03 ng/mL	Simple, fast and selective	Antibody-based detection may be affected by cross-reactivity; relatively broad recovery range	[10]
ELISA	Human saliva	<2 pg/mL detected in daytime saliva	Suitable for detecting low daytime MLT concentrations; high sensitivity	Commercial immunoassay kit required; antibody-based assays may be affected by cross-reactivity; requires laboratory immunoassay workflow	[11]
CV (GPH-CSPE)	Pharmaceutical products	1–300 µM/0.87 µM	Excellent sensitivity for voltammetric and amperometric detection of MLT; small amount of sample; low cost	LOD demonstrated in pharmaceutical products rather than biological matrices	[12]
SWV (G/SPE)	Pharmaceutical products	0.2–333.33 µM/0.34 µM	Simple, rapid and potentially portable; graphene provides high electroactive surface; low cost	Relatively low LOD compared with some previously reported electrochemical sensors; lack of biological recognition	[13]
DPV (MIP-PTBO/MWCNTs/GCE)	Human serum	1–1000 µM/0.027 µM	High sensitivity and selectivity; wide linear range; low influence of interfering species; molecular imprinting provides selective recognition	Requires multi-component electrode fabrication and molecular imprinting; involves nanomaterial/polymer synthesis and electrode modification; performance depends on optimized surface preparation and experimental conditions	[14]
SWV (AB-C/GE)	Synthetic blood serum	20–600 µM/ 1.81 µM	Relatively simple and rapid electrochemical method; demonstrated applicability in a biological matrix	LOD higher than that of more sensitive modern sensors; artificial blood serum rather than real human biological samples	[15]
DPV (HRP/G/SPE, enzymatic biosensor)	Pharmaceutical products	0.4–3.6 µM/1.02 µM	First enzymatic biosensor for MLT detection; high sensitivity; low-cost and simple platform; provides a basis for further development of enzyme-based biosensors for MLT; good LOD	Applicability was demonstrated only in pharmaceutical products	This work

**Abbreviations**: LC-MS/MS—Liquid Chromatography Tandem Mass Spectrometry; ELISA—Enzyme-Linked Immunosorbent Assay; CV—Cyclic voltammetry; SWV—Square wave voltammetry; DPV—Differential pulse voltammetry.

**Table 2:** Enzymatic kinetic parameters of the modified HRP/G/SPE biosensors.

Biosensor	I_max_ (μA)	$K_{m}^{app}$(μM)
R10–HRP/G/SPE	1996	4.15
R20–HRP/G/SPE	1948.17	2.91
F10–HRP/G/SPE	2000	2.63
F20–HRP/G/SPE	1870.55	1.16

**Table 3:** Comparison of $K_{m}^{app}$ values of HRP-based biosensors for the detection of different analytes.

Biosensor	Analyte	$K_{m}^{app}$	Reference
HRP–Chit/PL–LEU/PDA/GCE	H_2_O_2_	0.12 mM	[46]
Au/GS/HRP/CS	H_2_O_2_	2.61 mM	[47]
MWCNT-COOH-GABA/HRP/GCE	H_2_O_2_	0.23 mM	[44]
Hb–PpPDA@Fe_3_O_4_/GCE	H_2_O_2_	0.088 mM	[48]
HRP–MWCNTs–SiSG/Poly (Gly)/CPE	Dopamine	0.5 mM	[27]
HRP/G/SPE	MLT	1.16–4.15 µM	This study

**Table 4:** The influence of interfering compounds on the MLT detection.

Interfering Compound	Recovery (%)	RSD (%)	Tolerance Limit (M)
Serotonin	95.8	3.1	3.2 × 10^−5^
Dopamine	96.5	2.4	7.2 × 10^−5^
Uric acid	99.8	2.2	2.0 × 10^−3^
Alanine	99.1	1.9	5.2 × 10^−5^
Pyridoxine	97.2	2.6	3.8 × 10^−5^
Ascorbic acid	98.3	2.7	1.2 × 10^−4^
Glucose	97.9	1.8	1.6 × 10^−4^

**Table 5:** Experimental concentrations of pharmaceutical products obtained with HRP/G/SPE biosensors.

Pharmaceutical Product	Biosensor	I_theoretical_ (µA)	C_theoretical_ (µM)	I_experimental_ (µA)	C_experimental_ (µM)	Analytical Recovery (%)	Percentage Error (%)
Rotta Natura	R10	1765.8	0.7996	1765.14	0.7672 ± 0.0011	95.94	4.05
	R20	1761.27		1761.58	0.8186 ± 0.0013	102.38	2.38
	F10	1711.23		1712.41	0.8103 ± 0.0012	101.33	1.33
	F20	1755.06		1755.66	0.8576 ± 0.0014	107.24	7.24
Esi	R10	1765.8		1765.94	0.8329 ± 0.0012	104.16	4.16
	R20	1761.27		1760.85	0.7616 ± 0.0012	95.25	4.74
	F10	1711.23		1710.96	0.7471 ± 0.0009	93.43	6.56
	F20	1755.06		1754.87	0.7811 ± 0.0013	97.68	2.31
Cosmopharm	R10	1765.8		1765.52	0.7984 ± 0.0014	99.85	0.14
	R20	1761.27		1761.12	0.7827 ± 0.0012	97.89	2.11
	F10	1711.23		1711.85	0.7859 ± 0.0012	98.28	1.71
	F20	1755.06		1755.38	0.8305 ± 0.0015	103.85	3.85

## Data Availability

The authors confirm that the data supporting the findings of this study are available within the article.

## Abbreviations

The following abbreviations are used in this manuscript:

HRP	Horseradish peroxidase
MLT	Melatonin
G/SPE	Graphene-modified screen-printed electrode
DPV	Differential pulse voltammetry
CV	Cyclic voltammetry
SWV	Square wave voltammetry
LC-MS/MS	Liquid Chromatography-Tandem Mass Spectrometry
ELISA	Enzyme-Linked Immunosorbent Assay
POC	Point-of-care
R10-HRP/G/SPE	G/SPE functionalized with 10 µL of 5 mg/mL HRP, immobilized at room temperature
R20-HRP/G/SPE	G/SPE functionalized with 20 µL of 5 mg/mL HRP, immobilized at room temperature
F10-HRP/G/SPE	G/SPE functionalized with 10 µL of 5 mg/mL HRP, immobilized at refrigerator temperature
F20-HRP/G/SPE	G/SPE functionalized with 20 µL of 5 mg/mL HRP, immobilized at refrigerator temperature
LOD	Limit of detection
LOQ	Limit of quantification
